# Systemic Immunosuppression in High-Risk Penetrating Keratoplasty: A Systematic Review

**DOI:** 10.14740/jocmr2326w

**Published:** 2016-02-27

**Authors:** Shveta Bali, Richard Filek, Francie Si, William Hodge

**Affiliations:** a Department of Ophthalmology, Western University, London, Ont. N6A 4V2, Canada; b Department of Pathology, Western University, London, Ont. N6A 4V2, Canada; c Department of Epidemiology and Biostatistics, Western University, London, Ont. N6A 4V2, Canada

**Keywords:** Keratoplasty, High risk, Immunosuppression, Cyclosporine, Mycophenolate mofetil, Systematic review

## Abstract

Cornea transplantation has a high success rate and typically only requires topical immunomodulation. However, in high-risk cases, systemic immunosuppression can be used. We conducted a systematic review on the efficacy and side effects of systemic immunosuppression for high-risk cornea transplantation. The study population was 18 years old or older with a high-risk transplant (two or more clock hours of cornea vascularization or a previous failed graft or a graft needed because of herpes simplex keratitis). A comprehensive search strategy was performed with the help of an information specialist and content experts from ophthalmology. All study designs were accepted for assessment. Level 1 and level 2 screening was performed by two reviewers followed by data abstraction. Forest plots were created whenever possible to synthesize treatment effects. Quality assessment was done with a Downs and Blacks score. From 1,150 articles, 29 were ultimately used for data abstraction. The odds ratios (ORs) for clear graft survival in cyclosporine and controls were 2.43 (95% CI: 1.00 - 5.88) and 3.64 (95% CI: 1.48 - 8.91) for rejection free episodes. Mycophenolate mofetil (MMF) significantly improved the rejection free graft survival rates at 1 year (OR: 4.05, 95% CI: 1.83 - 8.96). The overall results suggested that both systemic cyclosporine and MMF improved 1-year rejection free graft survival in high-risk keratoplasty. Cyclosporine also significantly improved clear graft survival rates at 1 year; however, there were insufficient data to analyze the same in the MMF group. Higher quality studies are needed to understand this issue better.

## Introduction

Corneal transplantation is one of the most commonly performed human transplantation surgeries. The overall 10-year survival rates of corneal grafts range between 75% and 80% [1, 2]. However, in the presence of “high-risk” conditions, the survival rate drops to 30-50% at 3- to 5-year follow-up [3-5]. The Singapore Corneal Transplant Study revealed that while the 5-year transplant survival rate was as high as 100% for keratoconic cases, only 18% of corneas with regrafts survived at 5-year follow-up [2]. Bersudsky et al, in their study on repeat corneal grafts, noted that only 28% of the 78 first regrafts remained clear at the end of follow-up of 54 months [6]. The number dropped to 20% for subsequent regrafting [6]. The most common reason responsible for graft failure is immunologic rejection that accounts for almost one-third of the failure rates [2, 6, 7].

Ocular immune privilege has been described by Niederkorn as a “three legged stool” comprising an afferent arm blockade, deviation of the immune response to a state of immune tolerance and blockade of efferent arm [8, 9]. The afferent arm blockade results from avascularity, lack of lymphatics, low major histocompatibility complex expression and presence of native immunosuppression molecules. The clonal deletion, anergy and immune deviation serve as the central arm and protective molecules FasL and PD-L1 act to block the efferent arm of the immune cascade. A breach in any of the above increases the immunogenicity of the corneal transplant and places that cornea at “high risk” for immunologic rejection. The Collaborative Cornea Transplantation Study has described high-risk conditions as presence of more than two quadrants of corneal neovascularization and sensitization due to a previous graft [10, 11]. The other conditions that may place the cornea at a higher risk of rejection are position of the graft close to limbus [12], simultaneous limbo-keratoplasty, severe atopic dermatitis [13] and herpes simplex keratitis (HSV) [14, 15].

Reports from experimental models suggest that transported corneal alloantigens lead to clonal expansion of T cells in regional lymph nodes and spleen [16, 17], and have justified the use of systemic immunosuppression in high-risk cases in an attempt to prolong corneal graft survival. The common drugs included in these protocols are calcineurin inhibitors, including cyclosporine and tacrolimus, and antimetabolites, including MMF and azathioprine [18-21]. The calcineurin inhibitors bind to a specific cytosol protein and inhibit calcineurin-calmodulin-induced transcription of interleukin-2 and other early T-cell specific genes. MMF interferes with *de novo* synthesis of guanosine nucleotides by reversibly inhibiting the enzyme inosine monophosphate dehydrogenase.

A number of studies have been published to report the efficacy of these immunosuppressive agents as prophylaxis against corneal graft rejection [18-21]. However, the results have been inconsistent and there is lack of evidence-based guidelines about the use of these agents for immunoprophylaxis in high-risk corneal transplantation.

The purpose of this study was to conduct a systematic review of the published literature and examine the published evidence on efficacy of systemic immunosuppressive agents as prophylaxis against corneal graft rejection in cases at “high risk” of immunologic rejection after corneal transplantation.

## Methods

### Inclusion criteria

Any study investigating the role of systemic immunosuppression in high-risk penetrating keratoplasty was included in our review to maximize interpretability and generalizability. Patients had to be adults over 18 years of age. High risk was defined as two or more vascular quadrants in the cornea, surgery for a previously failed graft or a history of HSV keratitis in the operated eye.

### Study identification and databases

The search strategy for this project was comprehensive and was tailored to achieve the highest possible recall of relevant studies. An electronic search strategy was developed by an information specialist in consultation with two clinical content experts in corneal transplantation. The following bibliographic databases were searched: PubMed; Ovid’s Medline in-Process & Other Non-Indexed Citations, Medline, EMBASE and CINAHL; Thomson’s Social Sciences Citation Index and Biosis Previews.

Searches were not restricted by publication type, or study design. The search had no initial time restriction. The final search time was March 2014 but updates continued monthly until August 2014.

### Study design

Published and unpublished reports of any design were included for our systematic review. The review included studies with a comparison group (randomized controlled trial (RCT)), non-controlled and observational studies (pre- versus post-, prospective cohort, and case series designs).

### Study selection

We drafted specific screening questions for all levels of relevance assessment (level 1: title and abstract screening; level 2: full text relevance screening) and performed a calibration exercise involving questions developed specifically for this review. All records were uploaded into an internet-based, secured, software program (evidence for policy and practice information (EPPI)) to help manage the review. All records retrieved through searches were initially screened broadly (level 1) using titles, and abstracts and done by two reviewers. All records that were tagged at this level as a review article, report, or statement were screened for relevance for our review for reference matching. Reference lists of reviews that were thought to be relevant were screened for potentially relevant publications using reference list checking. Discrepancies were addressed by consensus between the two reviewers and when this was not possible, an adjudicator (WGH) resolved the conflict.

All studies identified as potentially relevant were retrieved in full-text format, and screened independently (level 2), again by two reviewers. The same method to address reliability and discrepancies was used as in level 1. All studies excluded at this level were placed in an excluded database, and exclusionary reasons were noted, and used to create a PRISMA (preferred reporting items for systematic reviews and meta-analysis) checklist.

### Data abstraction

Following a calibration exercise involving two studies, data abstraction was performed using an electronic data abstraction form developed and tailored specifically for this review. Study and outcome characteristics to be extracted included report (e.g., publication type, location, year of publication), study (e.g., sample size, research design, number of study arms/groups, cohorts, or phases), population (e.g., age, gender, diagnosis description, high-risk characteristics, duration of follow-up), treatment under study outcomes (e.g. type, dosage and duration of immunosuppressive agent, clear graft survival at 1 and 3 years, rejection free graft survival at 1 and 3 years), and adverse events (e.g., side effects, post-keratoplasty complications, systemic illnesses associated with the medication, withdrawals or termination of treatment). Data abstraction occurred with two reviewers, a primary reviewer and a secondary reviewer who verified the work.

### Study quality

We used the instrument generated by Downs and Black for appraising quality [22]. The Downs and Black checklist for quality assessment was selected as it has been developed to use with both randomized and non-randomized studies and is recommended as being suitable for use in systematic reviews [23, 24].

### Summarizing the evidence

Qualitative data synthesis was used when outcomes were not amenable to quantitative synthesis. When quantitative synthesis was available but too heterogenous for meta-analysis, the data were summarized but not in a forest plot. Quantitative data synthesis studies of the association between treatment option and outcome were considered for meta-analysis. The measure of association used was the odds ratio (OR) adjusted for possible confounding factors and was combined across studies where possible. For forest plots presented, only studies with a comparison group were used (i.e. case series were not used).

## Results

**Figure 1:**
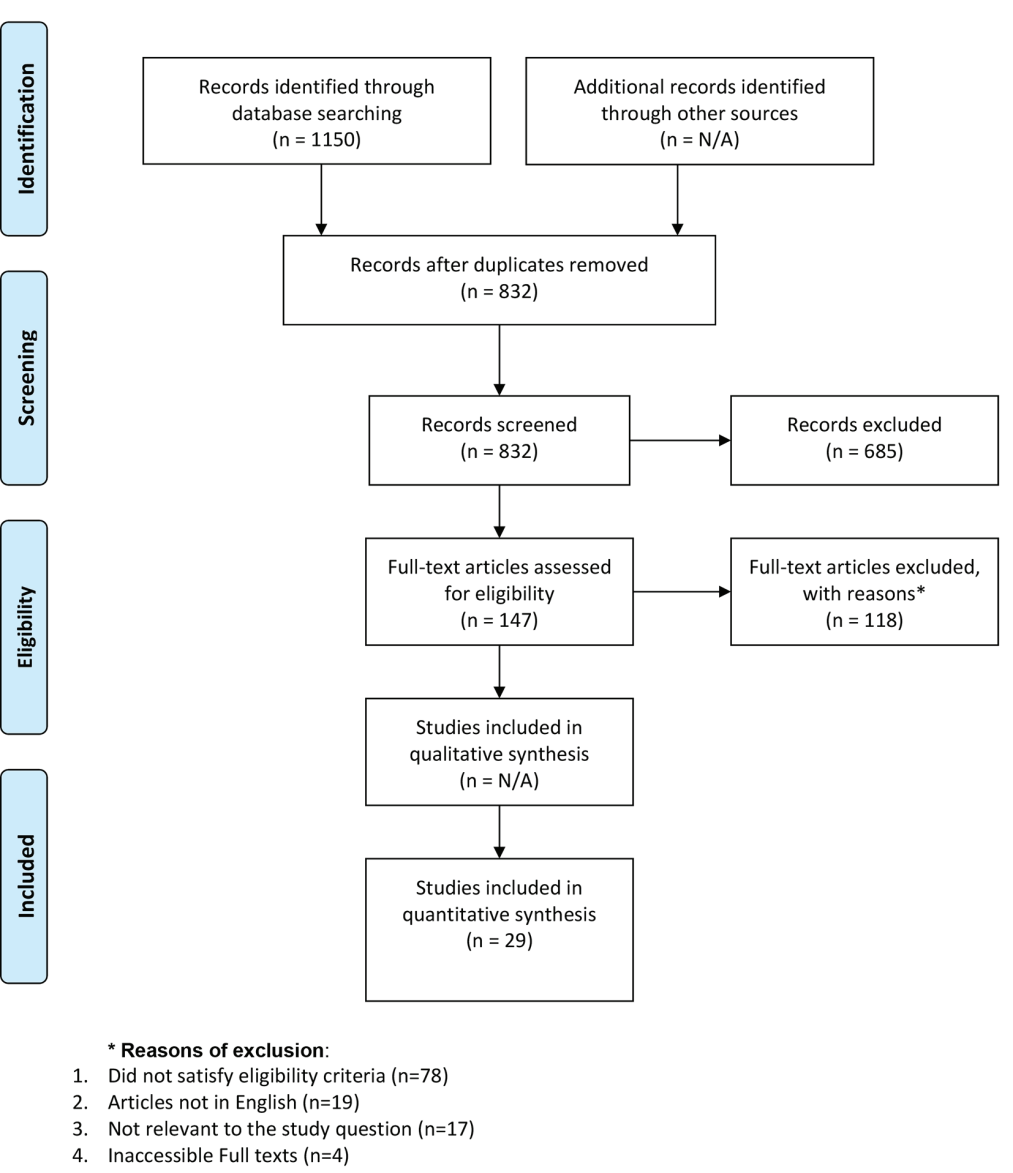
PRISMA diagram.

Figure 1 shows the PRISMA diagram for this review. A total of 1,150 studies of potential interest were identified by the original literature search. Two hundred sixty-eight articles were duplications and were eliminated. After screening the titles and abstracts, 735 further articles were excluded. For the remaining 147 articles, the full texts were retrieved and screened. A further 78 articles were excluded from the review based on this screening. Of the remaining 69 articles, 40 were excluded either because the full texts articles were inaccessible (n = 4), or the articles were not in English (n = 19), or were not relevant to the review (n = 17). Thus, a total of 29 papers were selected for the final analysis.

Of the 29 articles selected, eight were RCTs, 10 were cohort studies and 11 were case series. Sixteen studies evaluated the results of systemic immunosuppression cyclosporine A (CsA) alone, of which three were RCTs [18, 21, 25-37]. Four studies investigated the outcomes of high-risk grafts after immunosuppression with MMF alone and involved three RCTs [19, 38-40]. Four studies conducted a comparative evaluation of CsA and MMF and two of these studies were RCTs [15, 41-43]. The immunosuppression regimens used in other studies were rapamycin alone (n = 1) [44] or in combination with MMF (n = 1) [45], tacrolimus (n = 4) [20, 46-48], and azathioprine (n = 1) [21].

The quality assessment of the articles was done using the Downs and Blacks score [22]. The median score for all studies was 18, with a range from 6 to 24. Overall, the mean age of study participants was 41.73 ± 22.74 years. The mean percent of females for all the studies was 45.3%. The average follow-up period for all the studies was 25.45 ± 11.16 months.

At level 1 screening where 882 articles were screened, there were 25 disagreements for a kappa score of 0.90. At level 2 screening, there were 147 articles screened and there were 15 disagreements for a kappa score of 0.71.

### CsA

Sixteen studies evaluated the results of systemic immunosuppression in high-risk keratoplasty with CsA alone, of which eight were cohort studies, five were case series and three were RCTs.

The total number of subjects enrolled in all studies using cyclosporine was 817, of which 35.7% were females. Five hundred eighteen patients received systemic cyclosporine for post-keratoplasty immunosuppression (there were 299 controls) with the average blood CsA concentration ranging from 210 to 395 ng/mL. The mean age of patients included for studies was 49.5 ± 12.4 years. The mean post-operative follow-up period was 26.5 ± 12.9 months. The mean rejection free and clear graft survival rates at 1 year were 80.5±12.1% and 85.3±14.4%, respectively. Only six studies evaluated the long-term Kaplan-Meir survival rates at 3 years. In these studies, the mean rejection free and clear graft survival rates at 3 years were 67.6±16.4% and 54±26.8%, respectively. In patients on systemic CsA, 66.4% of the rejection episodes were successfully reversed with medical management. On the other hand, among control subjects, the average rate of rejection reversal was 27.8% (P = 0.02).

We found three RCTs comparing the efficacy of systemic cyclosporine against controls for immunosuppression following high-risk keratoplasty. Den et al evaluated 38 patients with high-risk keratoplasty, of which 57.9% were females [26]. Systemic CsA was used at a serum concentration of 500 - 800 ng/mL for at least 12 months. They did not find any significant difference in the clear graft survival rates between the cases and controls. Sanchez et al conducted an RCT wherein four patients received oral prednisone and CsA each for a period of 6 months following high-risk keratoplasty [21]. At a follow-up of 12 months, 100% grafts in the CsA group were rejection free, whereas in the control group, the rejection free graft survival was 0%. In another study, Shimazaki et al enrolled 39 patients in an RCT, 20 of which received systemic CsA (Serum CsA concentration: 600-1000 ng/mL) for prophylactic immunosuppression [34]. It was found that cyclosporine failed to show a positive effect in preventing rejection in high-risk corneal transplantation (1-year graft survival rates of 94.7% for CsA group and 94.1% for the control group).

**Figure 2:**
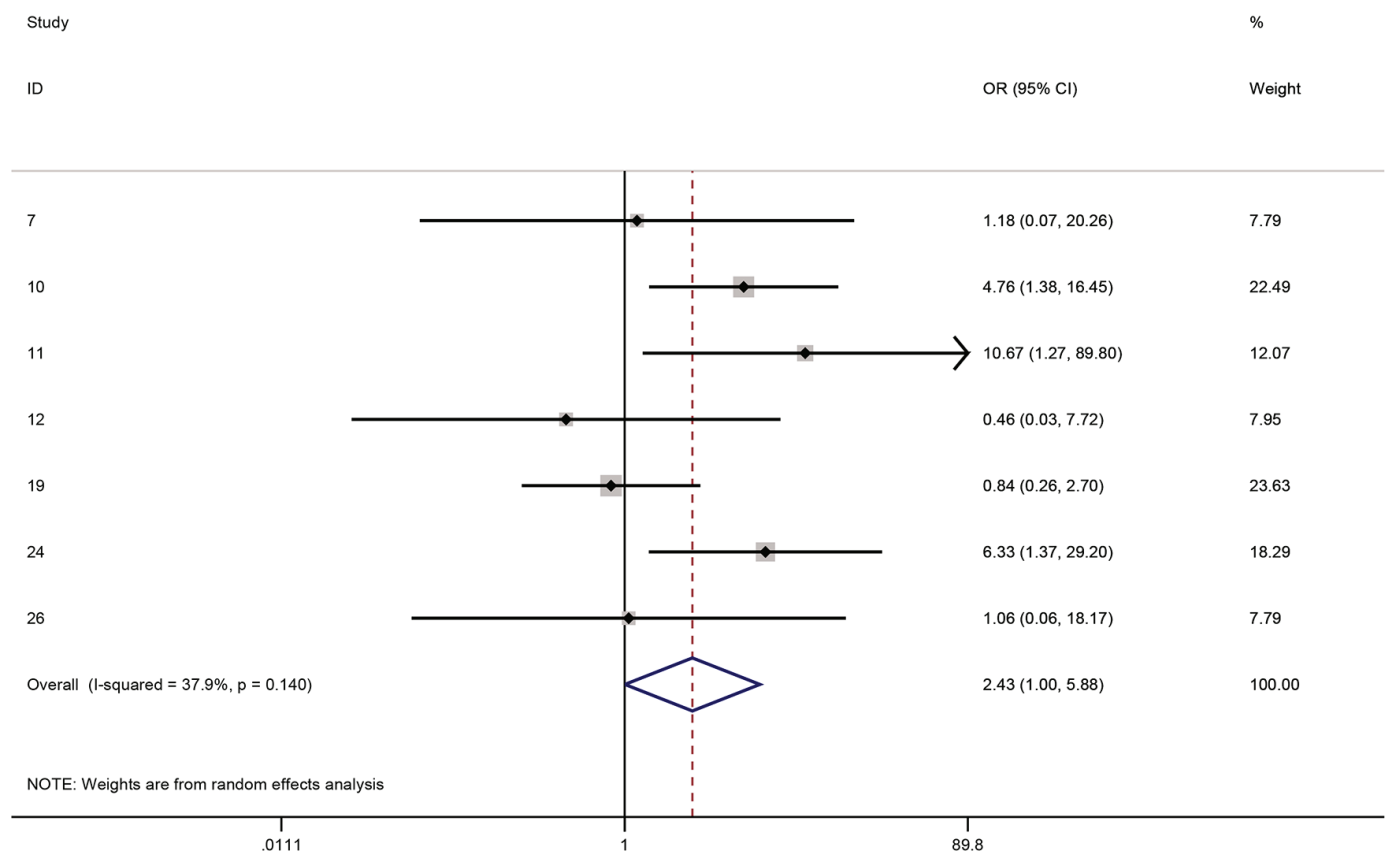
One-year clear grafts OR for cyclosporine vs. controls.

**Figure 3:**
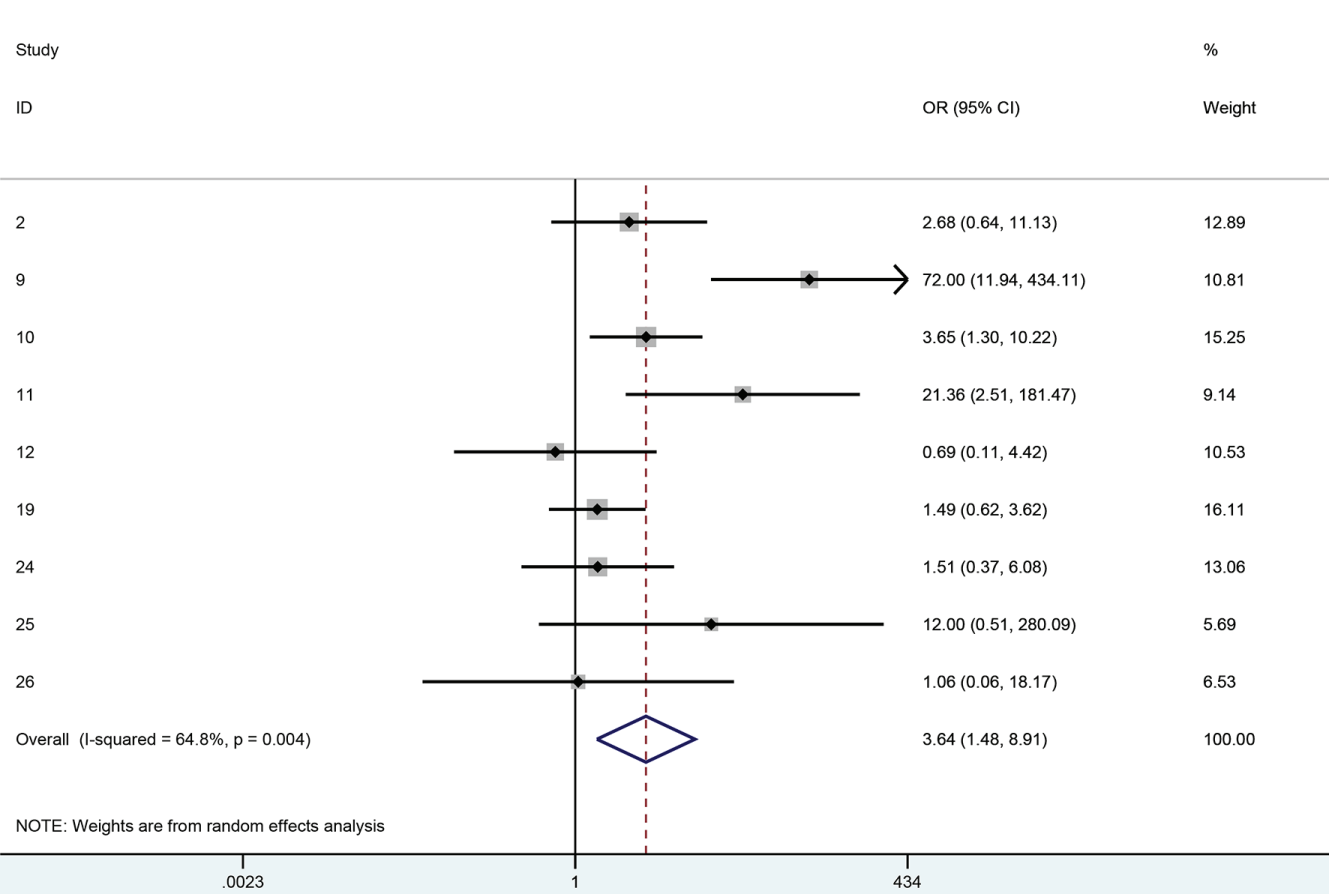
One-year rejection free OR for cyclosporine vs. controls.

Figures 2 and 3 show the forest plots for 1-year clear graft survival and 1-year rejection free episodes amongst all of the analytic studies (i.e. RCTs plus observational studies). Although there were 11 analytic studies in total, only 10 had 1-year data or longer. Six of the 10 studies reported both clear graft survival and rejection free episodes and were included in both forest plots, whereas four studies only reported one of the outcomes and hence were only found in one or the other forest plot. The OR for clear graft survival in cyclosporine vs. controls was 2.43 (95% CI: 1.00 - 5.88, I^2^ = 37.9%) and for rejection free episodes was 3.64 (95% CI: 1.48 - 8.91, I^2^ = 64.8%). We performed a meta-regression to see if any covariates significantly affected this relationship and studied age, gender, diagnosis, topical steroid duration, systemic steroid duration, study quality, and study design. No variable had a significant effect in the meta-regression. The Horbold-Egger bias was used to detect funnel plot asymmetry and publication bias was not found (P = 0.41).

### MMF

Four studies evaluated the results of systemic MMF on graft survival rates for patients undergoing high-risk corneal transplantation, and three of the studies were RCTs.

The total number of subjects enrolled in the four studies was 242, of which 147 received systemic MMF for prophylactic immunosuppression (95 were control subjects). MMF was used at a dosage of 2 g/day for 6 months in three studies, whereas one study used it for 12 months. The mean age of the participants was 56.95 ± 2.26 years and the mean follow-up duration was 25.9 ± 12.32 months. Overall, the mean Kaplan-Meir rejection free graft survival rate was 89.05% at 1 year. Only two studies reported the 3-year graft survival rate, with a mean of 76.5%. In patients on systemic MMF, the rate of reversibility of rejection episodes was 91.7%. This was higher as compared to the control group where only 52.05% rejection episodes were reversible (P = 0.01).

Three of these studies were RCTs. Birnbaum et al enrolled 98 of 140 scheduled patients after an interim evaluation showed a statistically significant result [19]. They noted that 83% of patients in the MMF group and 64.5% in the control group were free of immunologic rejection at a follow-up of 39.5 months (P = 0.044). Mayer et al evaluated 30 patients who underwent penetrating keratoplasty for herpetic eye disease [38]. Ten of these patients received MMF for 1 year for prophylaxis against immunologic reaction. They found that 90% of patients on MMF and 80% of controls were free of rejection at 36 months follow-up. This difference was found to be statistically significant. Reinhard et al conducted a randomized multicenter trial and published preliminary results of 86 patients [39]. Forty-eight of these patients received MMF for immunoprophylaxis and 89% of these grafts had no immune reaction at 1-year follow-up. This was noted to be significantly higher than the controls, where the 1-year rejection free graft survival was 67%.

**Figure 4:**
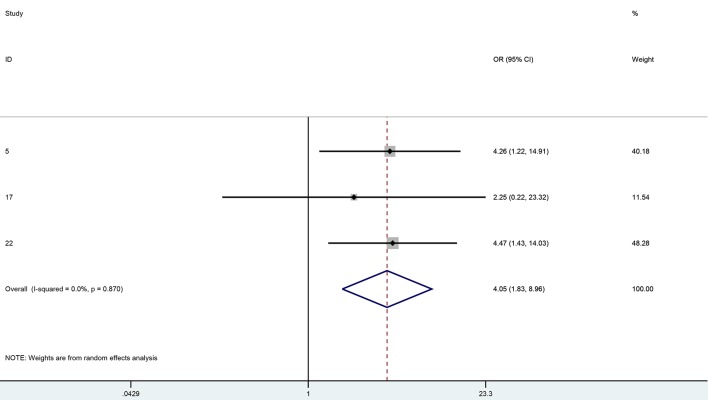
One-year rejection free OR for MMF vs. controls.

Forest plot results (Fig. 4) showed that systemic MMF significantly improved the rejection free graft survival rates at 1 year in high-risk keratoplasty (OR: 4.05, 95% CI: 1.83 - 8.96, I^2^ = 0%) There were not enough data to analyze the clear graft survival rates in this group. Meta-regression failed to show significant effect of any of the variables on graft survival or immunologic rejection outcomes. Studied covariates included age, gender, topical steroid duration, systemic steroid duration, study quality or study design. The Horbold-Egger bias was used to detect funnel plot asymmetry and publication bias was not found (P = 0.31).

### Cyclosporine versus MMF

Four studies compared the efficacy of CsA and MMF in promoting graft survival in high-risk penetrating keratoplasty. The total number of patients enrolled in these studies was 598. Of these, 304 received CsA, 278 received MMF and 16 patients received combined CsA and MMF. The mean age of study participants was 57.5 ± 4.02 years and the mean follow-up was 24.3 ± 12.7 months. In the cyclosporine group, at 1 year, the mean rejection free and clear graft survival rates were 88.8% and 88.6%, respectively. The respective mean rates for MMF patients were 88.6% and 97.3%. Overall, 88% of immune reactions in the CsA group and 81.3% in the MMF group were successfully reversed.

Two of these studies were RCTs. In both the trials, no significant difference was noted in the efficacy of CsA and MMF in prevention of graft rejection in high-risk penetrating keratoplasty [42, 43].

### Safety

Overall, nephrotoxicity was the most common adverse effect reported with the use of CsA and was noted in an average of 5.5% patients. The other reported significant side effects were gastrointestinal (4.3%), elevated blood pressure (4.02%), neurotoxicity (1.4%), skin rash (0.7%), and systemic infection (0.5%). For all studies, in 6.3% patients, CsA had to be withdrawn as a result of significant toxicity.

The most common adverse effect reported with MMF was gastrointestinal, reported in 13.6% of individuals. The other significant reported side effects were elevated blood pressure (9.2%), systemic infection (6.5%), hyperlipidemia (5.2%), hepatotoxicity (2.2%), tachycardia (2.1%), lymphoma (1.8%), and arthralgia/myalgia (1.2%). Overall, MMF had to be withdrawn in 10.5% of patients as a result of these adverse effects.

## Discussion

The overall results suggest that the use of both systemic cyclosporine and MMF improves 1-year rejection free graft survival in high-risk keratoplasty. CsA also significantly improved clear graft survival rates at 1 year; however, there were insufficient data to analyze the same in the MMF group. The use of both drugs was associated with higher reversibility of rejection episodes as compared with controls. The other drugs used in different studies were rapamycin, tacrolimus and azathioprine, but there were insufficient data to perform an accurate synthesis.

The safety profile of the systemic drugs was acceptable. The most common toxicity noted with CSA was nephrotoxicity followed by gastrointestinal and hypertension. MMF use was most commonly associated with gastrointestinal upset and hypertension. Overall, the medications had to be withdrawn in less than about 11% of patients due to toxicity.

The meta-analysis performed on MMF rejection free efficacy showed no heterogeneity among the small number of studies found. There was moderate heterogeneity for the meta-analysis performed on clear graft survival for cyclosporine (I^2^ = 37.9%). There was large heterogeneity found in the meta-analysis for cyclosporine rejection free survival (I^2^ = 64.8%). This was likely from two studies with very high ORs. Removing these studies from the meta-analysis produced results with a smaller OR but in the same direction as the full meta-analysis.

Overall, there were some lacunae in the data available pertaining to this research question. Firstly, the overall quality assessment of the studies included was average.

Of the 29 articles selected, only eight were RCTs. Eleven studies were case series and hence did not have a control group. The mean quality assessment score of the studies using Downs and Blacks instrument was 18, out of a possible total of 30. Secondly, there were insufficient follow-up data and results to conduct a meta-analysis on the effect of the drugs on long-term graft survival.

Also noteworthy is that while cyclosporine showed an overall beneficial effect on rejection and survival in analytic studies, it had a surprisingly null effect on both outcomes in the two larger RCTs [26, 34] except for one very small RCT [21]. This is an important point in that RCTs represent our highest level of evidence and the discrepancy between the RCT results and overall results indicate that these questions need to be studied further.

To conclude, the results of this systematic review demonstrate that both CSA and MMF have a clinical benefit in improving 1-year graft survival in patients undergoing high-risk keratoplasty. The choice between the two may be dictated by cost and availability, presence of co-morbidities and the clinical experience of the prescribing physician. More high quality studies are needed to understand this important issue better.
